# Turbine Passage Design Methodology to Minimize Entropy Production—A Two-Step Optimization Strategy

**DOI:** 10.3390/e21060604

**Published:** 2019-06-18

**Authors:** Paht Juangphanich, Cis De Maesschalck, Guillermo Paniagua

**Affiliations:** 1 School of Aeronautical Engineering, Purdue University, West Lafayette, IN 47906, USA; 2 School of Mechanical Engineering, Purdue University, West Lafayette, IN 47907-2088, USA

**Keywords:** optimization, turbine, turbomachinery, design

## Abstract

Rapid aerodynamic design and optimization is essential for the development of future turbomachinery. The objective of this work is to demonstrate a methodology from 1D mean-line-design to a full 3D aerodynamic optimization of the turbine stage using a parameterization strategy that requires few parameters. The methodology is tested by designing a highly loaded and efficient turbine for the Purdue Experimental Turbine Aerothermal Laboratory. This manuscript describes the entire design process including the 2D/3D parameterization strategy in detail. The objective of the design is to maximize the entropy definition of efficiency while simultaneously maximizing the stage loading. Optimal design trends are highlighted for both the stator and rotor for several turbine characteristics in terms of pitch-to-chord ratio as well as the blades metal and stagger angles. Additionally, a correction term is proposed for the Horlock efficiency equation to maximize the accuracy based on the measured blade kinetic losses. Finally, the design and performance of optimal profiles along the Pareto front are summarized, featuring the highest aerodynamic performance and stage loading.

## 1. Introduction

Turbomachinery aerodynamic design and optimization are vital towards guaranteeing the reduction of specific fuel consumption while maximizing the power output of future gas turbine engines [1]. The initial conceptual design of turbomachinery components often relies on expertise and design guidelines from previous engine programs. Once the overall architecture is decided, simplified 1D models are used to set the velocity triangles throughout the different stages. Subsequently, 2D throughflow solvers are adopted to determine the radial distribution of the flow quantities, relying on correlations and empirical models from experimental campaigns and historical engine data. Eventually, 2D blade profiles are parameterized in the cascade plane and radially stacked to generate full 3D turbine airfoils, including lean or sweep distributions [2]. A comprehensive 3D optimization approach is essential to obtain robust blade shapes and allow an exploration of a wide design space and reveal novel optimal trends that can help future stage designs. On the one hand, a proper parametrization strategy ensures a high degree of geometrical flexibility, while using as few design parameters as possible. On the other hand, the choice of the appropriate optimization method will determine the convergence rate towards the Pareto front in a timely and computationally inexpensive matter as possible. 

Researchers have used Bezier curves [3] or a combination of splines and b-splines to design airfoil geometries. B-splines have been proven to be a successful tool in reducing turbomachinery loss when coupled to an optimizer. However, they have limitations. Shelton et al. [4] stated that it was difficult to induce large changes to the stagger, trailing edge wedge and leading edge angle using b-splines. They instead used b-splines as a tool to fine-tune the design of the blade. Ghaly and Mengistu [5] used Non-Uniform Rational B-Splines (NURBS) to optimize an existing turbine airfoil design in 2D—NURBS are similar to B-splines except their control points each have a weight. Their parameterization showed that NURBS required fewer points to parameterize a compressor airfoil [6], as opposed to a turbine blade [7] due to the increased curvature of the suction side. Shelton et al. [3] optimized a turbine blade under transonic conditions incorporating a stacking and sweep law. They similarly observed that it was difficult to make large changes to the stagger, wedge angle, and suction side surface angles in their parameterization using b-splines. Hasenjäger et al. [8] adopted b-splines to optimize a low aspect ratio stator blade in 3D and encountered difficulties as well, attempting to limit the number of control points needed to represent the blade surface using spline-like strategies. 

Bezier curves have been applied in a wide variety of turbomachinery applications because they offer designers the prospect of using less parameters while controlling the curvature by removing the need to parameterize the knot vector [9,10]. Goel et al. [11] used this type of curves to define 2D turbine airfoils using 10 and 8 parameters for the suction and pressure side respectively. Pierret and Van den Braembussche [12] employed a series of Bezier curves to model the suction and pressure sides in segments. However, special care needs to be taken to ensure a proper continuity of the geometrical derivatives. Paniagua et al. [13] used Bezier curves to design and optimize four rows of a contrarotating turbine. Sousa and Paniagua [14] adopted these curves to optimize the design of supersonic turbine blades, illustrating the flexibility to generate geometries going from slender compressor blades, to thick turbine blades with high turning. Thorn and Hartfield [6] used a combination of Bezier curves to define 2D airfoil shapes, and NURBS to design the 3D blade with a total of 136 design parameters to represent the turbine stage.

The choice of optimization strategy will affect the rate of convergence to an optimal design. The majority of the turbine optimization efforts use a form of evolutionary algorithms [15], occasionally assisted by a surrogate model to provide a fast, though lower fidelity, performance evaluation. The main advantage of these strategies is their ability to find global minima without the risk of being trapped inside a local minimum. A popular strategy is the use of differential evolution, a subset of the evolution algorithms, originally developed by Price and Storn [16]. Authors such as Ghaly and Mengistu [5], Shelton [4], Hasenjäger [8], and Sousa [14] used a single objective optimizer. Ghaly and Mengistu opted for simulated annealing compared to Shelton who adopted a combination of hill climb and genetic algorithms. Thorn and Hartfield [6] also used a pure genetic algorithm.

The objective of this paper is to demonstrate a methodology for designing a turbine stage from a 1D mean-line analysis directly to a full 3D optimized design of the stator, rotor, and channel. This manuscript uses design requirements from the Purdue Experimental Turbine Aerothermal Lab (PETAL) [17] as a starting point for the 1D design.

## 2. Methods

### 2.1. Strategy Overview 

**Figure 1:**
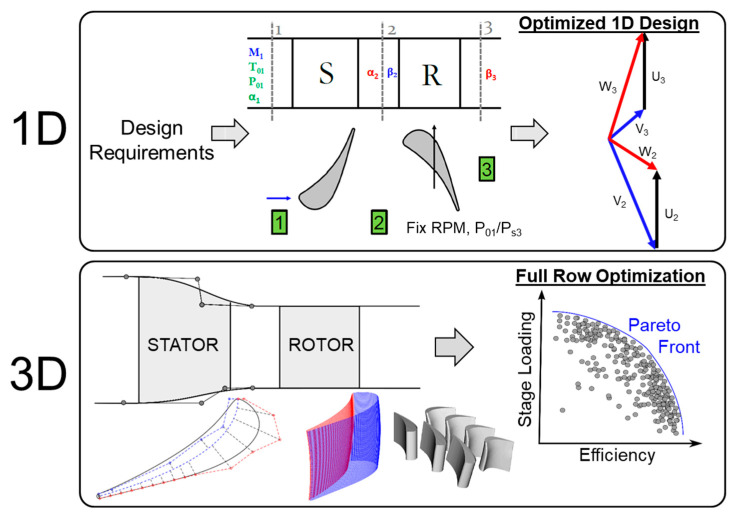
Overview of the overall entropy minimization strategy.

The design of a compact turbine stage for the Purdue annular wind tunnel consisted of 2 steps (Figure 1). In the first step, the facility geometrical requirements and a sweep of the facility’s operating regime were fed into a 1D multi-objective optimization routine. The objective of the 1D optimization was to find the ideal facility operating condition needed to produce a highly loaded efficient design. 

The 1D optimization provided the exit flow angles for the stator and rotor and the boundary conditions needed by the second step, 3D optimization. The design process for the 3D geometry consisted of passing a file containing a vector of parameters that is used to define the 2D profiles for the stator and rotor at different radii, and then stacking them to form a 3D blade. A lean distribution was applied individually to the blades. The blade profiles were then placed in between two end-walls, hub and shroud, which are also part of the optimization. The objective of the 3D design is to find geometries that maximize stage loading (ψ) and efficiency. 

### 2.2. 1D Optimization Methodology

The 1D stage optimization consisted of an in-house developed 1D MATLAB mean-line solver. The solver accepts inputs of a total pressure (P_01_) and total temperature (T_01_) at the inlet, rotational speed (RPM), total-to-static pressure ratio (P_01_/P_s3_), inlet Mach number (M_1_), and degree of reaction (r_p_). Geometrical constraints include inlet height (H_1_), and ratio of channel heights (i.e., H_2_/H_1_ and H_3_/H_2_). The inlet and outlet blade metal angles (α_2_ and β_3_) were iterated upon to balance the mass-flow through the stage. An axial inflow (α_1_ = 0), moderate stator and rotor efficiencies (respectively 92.5% and 87%), and constant gas properties were used. Constraints included an upstream total pressure of 5 bar and the rotational speed was fixed to 7500 RPM to maintain the structural integrity of the future rotating assembly. 

The optimization routine was paired with a multi-objective differential evolution optimizer to explore a large, 7-dimensional design space; which allowed the pressure ratio to vary from 3 to 5, the inlet Mach number from 0.1 to 0.3, the degree of reaction from 0.25 to 0.75, and the total inlet temperature from 500K to 700K based on the capacity of the upstream air heater. The remaining 3 degrees of freedom are the turbine inlet height which varied from 40mm to 63mm and the channel height ratios, H_2_/H_1_ and H_3_/H_2,_ varied from 1 to 2 and 0.5 to 1.5, respectively. The mean radius was constrained by the dimensions of the tunnel and was held at a constant of 389mm.

To exclude aerodynamically unfeasible designs, constraints were set for the flow angles and velocities based on prior turbine design knowledge. The passage height was restricted to 420 mm. The airfoil exit metal angles, α_2_ and β_3,_ were limited to 80 deg., the relative rotor inlet angle β_2_ to 40 deg., and a maximum allowed turning of 120 deg. in the rotor passage was imposed. To avoid high supersonic designs, the outlet Mach numbers, M_2_ and M_3r,_ were constrained to 1.3 and 1.05, respectively, while M_2r_ was kept below 0.42. Furthermore, mass-flow was limited to a maximum of 30 kg/s based on the capacity of the upstream high-pressure vessels and downstream dump tank. The target was to simultaneously maximize the stage loading (Equation (1)) and stage efficiency (Equation (2)).

(1) $$\psi =\frac{h_{01}-\;h_{03}}{U^{2}}$$

(2) $$\eta_{adiabatic}=\frac{{\dot{W}}_{shaft}}{h_{01}-h_{03ss}}=\frac{Torque*\omega }{Torque*\omega +T_{3}\left(s_{3}-s_{1}\right)}$$

This work uses as figure of merit, the adiabatic efficiency, specifically the ratio of extracted work to what would be isentropically possible with adiabatic boundary conditions. Therefore, the associated entropy production caused by heat transfer to the airfoils and end-walls can be neglected. In case isothermal simulations would be performed, the heat dissipated across the airfoils, hub and casing end-walls ought to be considered, by subtracting the energy lost through heat transfer from the actual drop in total enthalpy across the stage using Equation (3).

(3) $$\eta_{isothermal}=\eta_{adiabatic}+\frac{{\dot{Q}}_{stage}}{h_{01}-h_{03ss}}-\frac{T_{03}}{h_{01}-h_{03ss}}\left[\frac{{\dot{Q}}_{stator}}{T_{01}}+\frac{{\dot{Q}}_{rotor}}{\frac{1}{2}\left(T_{02}+T_{03}\right)}\right]$$

De Maesschalck [18], based on the work of Yasa et al. [19], and Atkins and Ainsworth [20] demonstrated the use of this correction to compare adiabatic with isothermal simulations, considering the temperature at which the heat transfer takes place for the entropy creation.

### 2.3. Three-Dimensional Optimization Strategy

**Figure 2:**
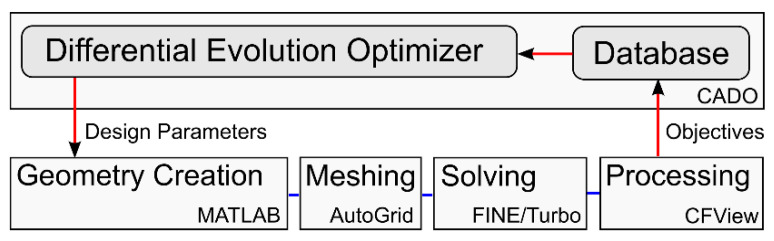
Overview of the 3D optimization structure.

The full 3D row optimization was implemented through an automatic evaluation routine written in BASH. The routine launched a MATLAB tool that created the 3D geometry using 75 parameters that defined the stator and rotor blades and hub and shroud curves. The 3D geometry is exported to a GeomTurbo file where it is read by the mesh generator, Numeca Autogrid. The mesh is solved using FINE/Turbo and post processed in CFView (Figure 2). The two optimization objectives: the entropy definition of efficiency (Equation (3)) and stage loading (Equation (2)) were extracted using Numeca CFVIEW and fed back into the optimizer. The entropy definition was used to reveal the effect of entropy on the overall turbine performance. 

### 2.4. 2D Airfoil Parameterization

**Figure 3:**
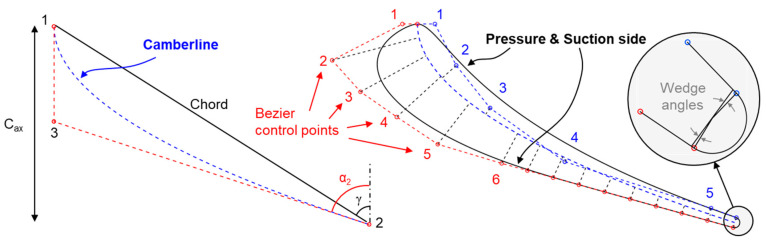
2D Parameterization strategy for the blade profiles in the 2D cascade planes.

The 2D blade was constructed in the radius, tangential, axial (RTZ) coordinate system. The 2D profile was defined using a camber-line and an independent parameterization of the suction and pressure sides. Compressor airfoils are usually defined with only a camber-line and one thickness distribution, with high flexibility and a lower number of variables. However, in transonic airfoils the local curvature of the rear suction side is particularly important, which requires an unconstrained shape for pressure side and suction side, which results in a few more parameters. Both the pressure and suction sides were defined using Bezier curves by assigning control points at distances perpendicular to the camber line (Figure 3-right). In the stator suction side, points 1-6 were spaced using an expansion ratio of 1.2, the points covered 60% of the camber-line. The remaining 40% of the camber-line is straightened out using 10 automatically spaced control points to maintain gradual diffusion. In the current transonic turbine design, the rear suction side was kept flat based on our design experience [21]. The camber-line is constructed by 3 points that define the inlet (α_1_, β_2_) and outlet (α_2_, β_3_) metal angles. The stagger angle (γ) and axial chord (C_ax_) determines the position of point 1. Throughout the optimization, the stator inlet flow angle, α_1_, was fixed to zero while the other blade metal angles, as well as the chord and stagger angle, could vary.

The pressure side was constructed from 5 control points (excluding the points at the leading edge and trailing edge) spaced using the same expansion ratio. The thickness of the point 1 on the pressure side was adjusted perpendicular to the metal angle to match the second derivative of the suction side at the beginning of the leading edge. The last point, 5, was fixed at 90% of the camber line. The thickness near the trailing edge along the pressure side was determined by the pressure side wedge angle. Finally, the trailing edge connected the two blade sides through a circle segment with a diameter of 1 mm.

Similar types of parametrization strategies have been successfully used in several prior studies and allows for the generation of a wide variety of turbine profiles [7,22]. This 2D parametrization gave rise to a total amount of 12 and 14 design variables for the stator and rotor, respectively. In the case of the stator, the blade profiles were parameterized at the hub and tip sections while the rotor included an additional profile at mid-span.

### 2.5. 3D Blade Parameterization

**Figure 4:**
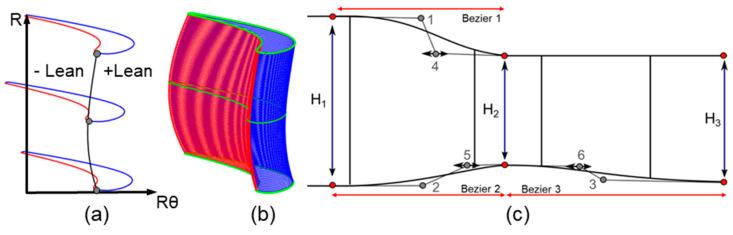
(**a**,**b**) Three-dimensional stacking of the blade profiles through a lean distribution. (**c**) Channel parameterization.

The 3D blade was constructed by stacking 2D profiles at fixed percent spans. Two stacking laws were defined: in the stator, stacking was done based on the leading edge; the rotor was stacked based on the centroid of the profiles. The two 2D profiles were used to define the 3D stator blade and three sections were used for the rotor. An example of the 3D stator and rotor designs are presented in Figure 4. After the profiles have been stacked, the next step is to smoothly blend them. This was accomplished by constructing 100 intermediate profiles along the spanwise direction. Splines were fitted in the spanwise direction through each point along the suction and pressure side of each profile allowing for the creation of the intermediate profiles. 

Once the 3D blade has been defined, blade lean was applied by constructing a Bezier curve from hub to tip through either the centroid or leading edge (Figure 4b). This determined the tangential shift of the intermediate profiles (Figure 4a). The Bezier curve was defined by 3 control points at the hub, midspan, and tip. The upper two points could move along the peripheral direction to induce lean by as much as 15% of the blade span. Positive lean is lean towards the suction side and negative lean is towards the pressure side. Figure 4b shows positive lean at the tip but negative lean at the midspan. In the final step, the 3D blade’s profiles are scaled radially to fit along streamlines in the channel. An inter-row spacing was one third of the axial stator hub chord as assumed. 

The strategy for the channel parametrization is presented in Figure 4c. The hub and shroud contours were constructed using 3 cubic Bezier curves and a straight line over the rotor shroud. Intermediate points 1, 2, and 3 were fixed axially at the stator and rotor mid-chords and were determined radially by the channel spans H_1_, H_2_ and H_3_. To control the local curvature, points 4, 5 and 6 could move axially from the profiles mid-chord all the way up to the vicinity of the stator-rotor interface. 

### 2.6. Computational Domain and Grid Sensitivity

**Figure 5:**
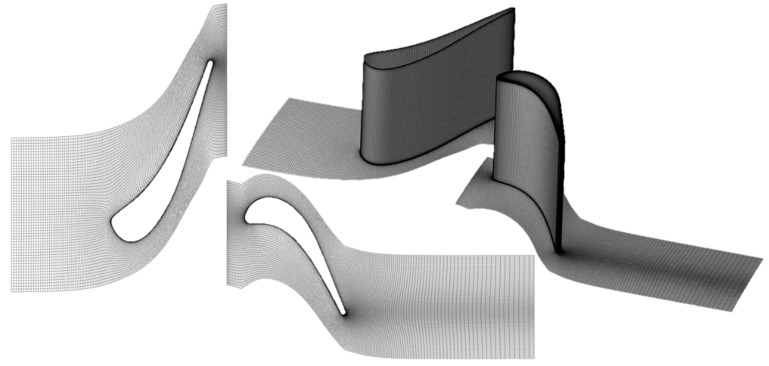
Computational domain.

Numeca Autogrid used an O4H structured topology to mesh the blades and construct the computational domain shown in Figure 5. Periodicity was applied to both the stator and rotor passages connected through a conservative coupling by pitchwise rows mixing plane. The stator mesh contains 117 spanwise cells while the rotor contains 141 cells. To account for tip clearance, 37 spanwise cells were defined within a rotor tip gap of 0.4mm. The stator contains 193 cells along the suction side and 97 cells on the pressure side. The rotor was discretized using 257 and 97 cells, respectively. The y+ was kept below unity using an initial cell size of 1 micrometer combined with an expansion ratio of 1.3 resulting in a total mesh count of 7.8 million cells. 

The computational domain was solved in Numeca FINE/Turbo using steady Reynolds-Averaged Navier-Stokes. Menter’s Shear-Stress Transport (SST) model was used for the turbulence closure [23]. The working fluid was dry air modeled as a real gas, incorporating temperature dependent properties of gamma, specific heat, and viscosity. Inlet total temperature and pressure were imposed while a fixed static pressure was set at the outlet. A turbulence intensity of 2.5% and length scale of 5% of the blade span were used for the inflow conditions. 

**Figure 6:**
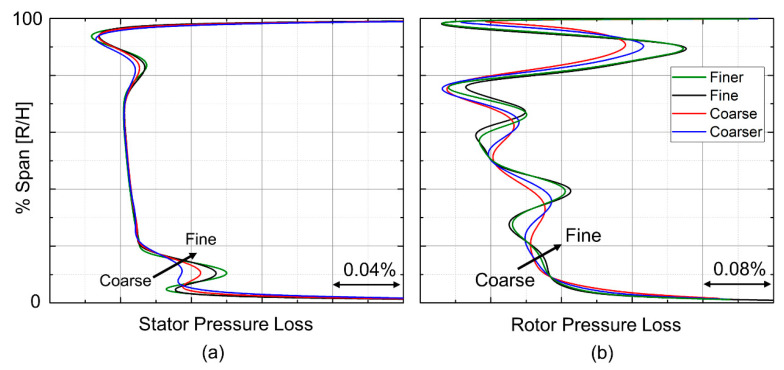
Stator and rotor pressure losses for four meshes of the grid sensitivity analysis. (**a**) Stator pressure loss; (**b**) Rotor pressure loss.

To select a grid size for the optimization, a mesh sensitivity study was performed separately for the stator and rotor using a baseline geometry. The stator mesh study was performed using the total pressure found in the 1D optimization and the static pressure given by the degree of reaction. Figure 6-left shows the stator-pressure loss along the span for the coarse to finer mesh. The fine mesh was selected for the stator due to its ability to accurately capture the pressure loss at the hub and tip. 

The rotor sensitivity study was performed by taking the exit boundary conditions from the stator and imposing it on the rotor mesh’s inlet. Outlet static pressure from the 1D simulation was used. Figure 6-right highlights the different mesh sizes and their impact on pressure loss along the span. The fine mesh was selected for the optimization because it matched the trend in pressure loss the best. Table 1 presents the details of the four different grids applying consecutive refinements in every direction.

**Figure 7:**
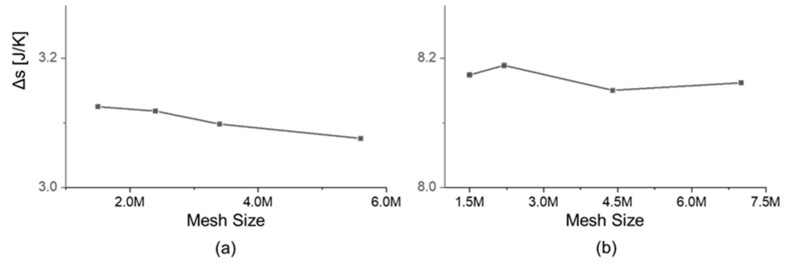
Effect of mesh size to the entropy creation across each row: (**a**) Stator; (**b**) Rotor.

In the calculation of efficiency, the authors considered the change of entropy across each blade row. The entropy change across the stator was evaluated as the difference between the values obtained at planes 1 and plane 2, the stator outlet plane (2) is located upstream of the mixing plane. At each plane, the values are the result of the integral along all the area, weighted by the local mass-flow in each cell, according to the method of Denton and Pullam [24]. The entropy change across the rotor was evaluated as the difference between the values obtained at planes 2’ and the rotor exit plane 3. The rotor inlet plane (2’) is located downstream of the mixing plane. Figure 7a shows the change of entropy across the stator. Figure 7a displays the change of entropy across the rotor, as a function of the grid size. The relative error in entropy for the fine mesh for the stator is 0.7% from the finest mesh. For the rotor, the relative error fine from finest mesh is less than 0.14%.

### 2.7. Optimization Setup

The optimization tool used for this project is CADO (Computer Aided Design and Optimization Tool). CADO was developed at the von Karman Institute for Fluid Dynamics [7]. It has been previously applied for the improvement of a wide variety of turbomachinery components [25,26,27]. CADO uses a multi-objective differential evolution strategy based on the Darwinian evolution [16], where every iteration a new set of individuals is generated via the process of crossover and mutation. These children are subsequently combined with the previous population through a NSGA-II [28] ranking. The population evolves over time through the entire design space, favoring the individuals with the highest efficiency and stage loading. Eventually the individuals converge to a Pareto front representing the subset of optimal profiles that maximize the two objectives. Due to the large amount of design variables, no surrogate model was used, and the performance of every individual was solely assessed using a high-fidelity CFD (Computational Fluid Dynamics) simulation. 

Table 2 summarizes the main optimization constants, namely the mutation scale factor and the mutation rate. Common values of the mutation rate vary from 0.5 to 1 [16,29]. Price et al. [30] recommends a crossover rate of 0.9 for functions with dependent parameters, and 0.5 for independent parameters. Gämperle et al. [31] showed that a large crossover rate speeds up the convergence, however convergence rate may decrease, or populations may approach a local minima and that a good choice is between 0.3 and 0.9.

Table 3 shows the evaluation space for both the 1D and 3D optimizations. The 1D optimization was seeded by evaluating 80 randomly selected individuals. Mutation and crossover were used on the parameters defining those 80 individuals to generate the next population with a size of 40 individuals. The recommended population size is 2 to 10 times the number of parameters [32]. The number of populations was determined by stopping the simulation once a pareto front was identified. The 3D optimization, on the other hand, had a large design space, and a fractional factorial [33] approach was used to initialize a database containing 256 individuals selected to cover 10% and 90% of the design space. From this set of individuals, the multi-objective optimization was started with a population size of 30 individuals. This population size facilitated a balance between a fast iteration turnover time and the ability to capture a sufficient amount of geometrical variability within each population.

### 2.8. Blade Count Selection

In the present study, the number of blades was not part of the optimization. Instead, several factors influenced the selection of the number of blades: The axial chord requirement for both stator and rotor blades could not exceed 45mm; the aspect ratio was limited by the inlet height from the 1D design. The strategy was to choose the number of blades while being able to explore a wide range of pitch-to-chord and aspect ratios. For this analysis, 41 stator and 61 rotor blades were chosen. This allowed the stator’s pitch-to-chord ratio to vary from 0.7 to 0.94, and aspect ratios (H/C) of 0.47 to 0.68. The rotor’s pitch-to-chord ratios ranged from 0.65 to 0.85 and aspect ratios of 0.7 to 1.2, simply by varying the axial chord and stagger angle of each blade.

## 3. Results

### 3.1. 1D Optimization Results 

**Figure 8:**
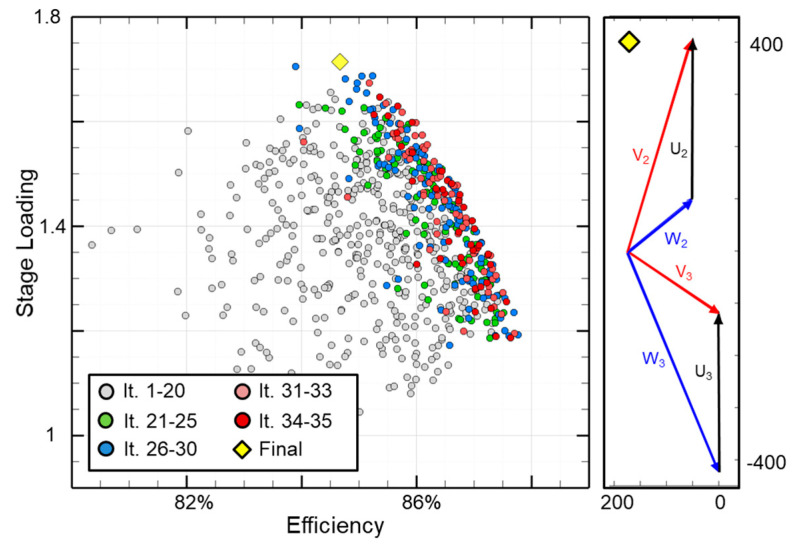
1D optimization results (**left**) and chosen design (**right**).

Figure 8-left presents the results of the 1D optimization—760 feasible designs were obtained from 35 optimization populations. The axes depict the two objectives: the simultaneous maximization of the aerodynamic efficiency and blade loading. The first 20 populations are depicted using grey markers, and are largely scattered around the design space. As the number of populations increases, the individuals coalesce towards the upper right where efficiency and stage loading are the highest. This is the pareto front.

The highest loaded design was chosen as the target configuration (indicated with a yellow diamond, Figure 8-right). This design shown in Table 4 consists of a stage loading of 1.74 satisfying all constraints while delivering a total power of 3.64 MW. The degree of reaction of the selected case is 0.39 with a pressure ratio of 4 and an inlet temperature of 676K. The turbine has a turning angle of 107 deg. while the stator outlet angle is 73 deg. Both the vane and blade exit Mach numbers, 0.89 (M_2_) and 1.04 (M_3r_), are in the high subsonic-transonic regime while both the stator and rotor passage heights are increasing, with 16% and 32% respectively. The pressure ratio, inlet total temperature will be used for the boundary conditions and the metal angles will be used to define a range for the following 3D optimization.

### 3.2. 3D Optimization Results 

#### 3.2.1. Pareto Front

**Figure 9:**
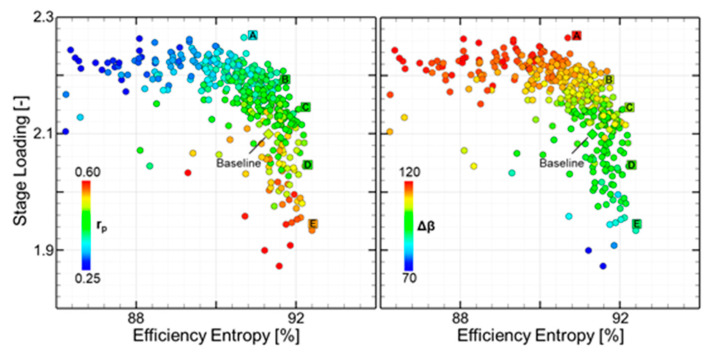
Pareto front (**left**) and colored by the degree of reaction and turning angle (**right**).

The 3D optimization ran for 15 generations (Figure 9-left). Each circle on the plot represents a unique combination of stator, rotor, and channel. Throughout the optimization, the individuals move to regions of high efficiency and stage loading. The pareto front represents the limit where if a geometry wanted to improve stage loading, a sacrifice in efficiency would have to made. Optimal designs along the Pareto front are indicated with letter A through E. The baseline profile is marked diamond. The individuals are colored by the degree of reaction (r_p_). On the right of Figure 9, the same design space is colored with the rotor turning angle (Δβ). Individuals with higher efficiency contain higher degrees of reaction and have lower turning. However, designs with higher stage loading, feature lower degrees of reaction and more turning. Degrees of reaction in the 0.3-0.35 range with turning up to 120 degrees represent the top right of the pareto front.

#### 3.2.2. Trends in Stator Loss Generation

**Figure 10:**
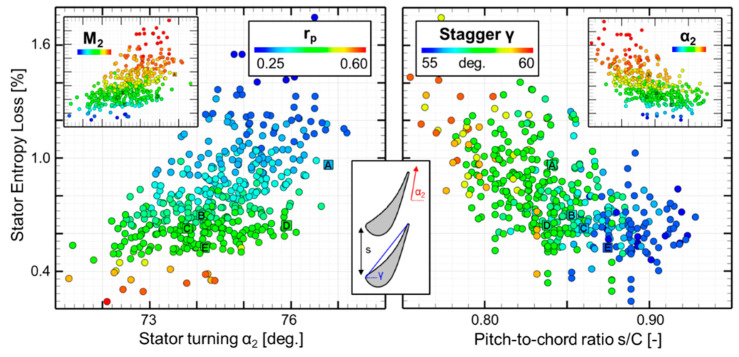
Relation of the stator losses with the aerodynamic quantities (**left**) and geometrical characteristics (**right**).

Figure 10 compares stator entropy loss coefficient [34] (Equation (4)) with stator turning (left) and pitch-to-chord ratio (right).

(4) $$\zeta_{s}=\frac{T_{2}\Delta s}{h_{02}-h_{2}}$$

The lower region of the Figure 10-left features designs of high degree of reaction, which results in less turning and lower exit Mach numbers (M_2_). The upper regions are characterized by low degree of reactions which results in more turning of the stator and have higher exit Mach numbers. Losses can be kept as low as 0.6% if one limits the stator turning to 76 deg. and a degree of reaction above 0.4.

Figure 10-right compares the entropy loss with geometry properties, pitch-to-chord ratio and stagger angle. Stators with lower loss have lower stagger angles closer to the minimum value and higher pitch-to-chord ratios. However, stators with higher turning have pitch to chord ratios below 0.85 to provide guidance, but they generate more loss. No clear optimal trends were observed in terms of lean distribution or suction side wedge angle for the simulated configurations.

#### 3.2.3. Trends in Rotor Loss Generation

**Figure 11:**
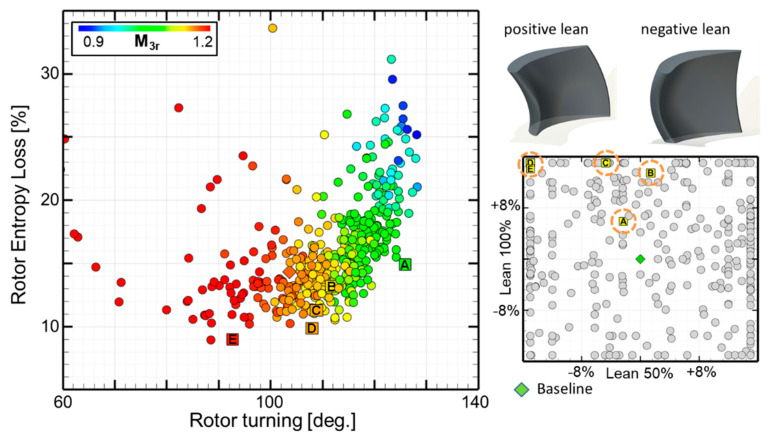
Effect of the rotor turning (**left**) and the influence of the blade lean (**right**).

Figure 11 presents the effect of several aerodynamic and geometrical features on the rotor entropy loss coefficient (Equation (4)). The left graph illustrates the effect of the rotor turning on the losses. The left portion of the graph shows low turning angles which results in high exit relative Mach numbers and as a consequence, a larger degree of reaction. This results in a lower incoming absolute flow velocity from the stator. Through a proper optimization of the rotor geometry, the losses can be limited to about 10% up to a turning angle of 110 deg. From this point on, as the turning increases, the losses start to increase significantly up to 15% for a turning angle of 125 deg.

The right graph of Figure 11 illustrates the lean distribution for the optimal profiles along the Pareto front, colored in yellow. The vertical axis identifies the lean at 100% span and in abscissa the lean at 50% span. The baseline design used in the grid sensitivity is marked with a green diamond showing no lean. The majority of the optimal profiles that display the best efficiency show positive lean at 100% span in order to off-load the tip section and negative lean in the mid-chord.

Similar to the stator, investigations on rotor loss with pitch-to-chord ratio contoured with the stagger angle γ was investigated. However, no apparent trend can be observed. It should be noted that the selected profiles along the Pareto front (A to E) are contained within a narrow band of the pitch-to-chord ratio of 0.7 to 0.75 and the majority of the profiles feature stagger angles between 40 and 45 degrees.

#### 3.2.4. Horlock Efficiency

Horlock efficiency, commonly used in turbomachinery to evaluate the efficiency of a stage from the losses of the stator and rotor, relies on a couple of simplifications. The first one, shown in Equation (5), is the assumption that v_3_ and v_3ss_ are equal, which however may result in errors for high speed turbines.

(5) $$\eta_{adiabatic}=\frac{Power}{Power+\left(h_{3}+\frac{v_{3}^{2}}{2}-h_{3ss}-\frac{v_{3ss}^{2}}{2}\right)}≅\frac{Power}{Power+\left(h_{3}-h_{3ss}\right)}$$

The enthalpy loss can be related to the difference in entropy using the second law of thermodynamics. Then h_3s_-h_3ss_ is related to h_2_-h_2s_ by the temperature ratio.

(6) $$h_{3}-h_{3ss}=\left(h_{3}-h_{3s}\right)+\left(h_{3s}-h_{3ss}\right)\;h_{3s}-h_{3ss}\;≅T_{3}\left(s_{3s}-s_{3ss}\right)\;h_{2}-h_{2s}≅T_{2}\left(s_{2}-s_{2s}\right)$$

(7) $$h_{3s}-h_{3ss}≅\frac{T_{3}}{T_{2}}\left(h_{2}-h_{2s}\right)$$

Substituting Equations (7) into (5) results in the definition of Horlock efficiency [35].

(8) $$\eta_{adiabatic,Horlock}=\frac{Power}{Power+\left(h_{3}-h_{3s}\right)+\frac{T_{3}}{T_{2}}\left(h_{2}-h_{2s}\right)}\;h_{2}-h_{2s}=\frac{V_{2}^{2}}{2}\xi_{stator}\;h_{3}-h_{3s}=\frac{W_{3}^{2}}{2}\xi_{rotor}$$

**Figure 12:**
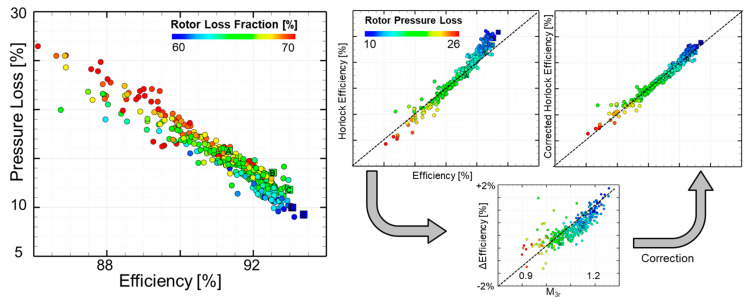
The stage efficiency in function of the aerodynamic losses (**left**) and the compensation strategy for the Horlock efficiency equation (**right**).

Figure 12-left shows the pressure loss of the rotor as a function of the entropy definition of efficiency as obtained using Equation (3), and the individuals are contoured with the percentage of rotor loss from the total loss of both blades. As the efficiency of the designs increase, pressure loss decreases, and the percentage of rotor loss from the total pressure loss reduces to 60%. The slope is approximately 3% of rotor loss for about 1% of stage efficiency gain. 

The middle plot in Figure 12 presents the comparison of the entropy efficiency (Equation (3)) with the efficiency approximated using the Horlock equation (Equation (8)). The Horlock equation is used in experiments and simulations to provide an estimate for the total efficiency of the stage, based on the measured kinetic loss of the flow. The kinetic loss for the stator was computed from the mass-flow average relative velocity, V_2,_ extracted at the exit of the stator (Equation (9)). Similarly, for the blade losses, the mass-flow average relative velocity, W_3,_ was taken at one half the axial chord downstream of the rotor (Equation (10)).

(9) $$\xi_{stator}=\frac{V_{2s}^{2}-V_{2}^{2}}{V_{2}^{2}}$$

(10) $$\xi_{rotor}=\frac{W_{3s}^{2}-W_{3}^{2}}{W_{3}^{2}}$$

Figure 12-right illustrates the comparison of both efficiency definitions. The corrected Horlock efficiency definition (Equation (11)) was evaluated on a dataset of 416 different designs, and the resulting error has a mean value of 0.01%, ±0.007% with a confidence level of 95% from the entropy definition of stage efficiency.

(11) $$\eta_{adiabatic,\;Horlock\;corr}=\eta_{adiabatic,\;Horlock}-0.0562\left(M_{3r}-1.057\right)$$

#### 3.2.5. Analysis of Optimal Profiles 

**Figure 13:**
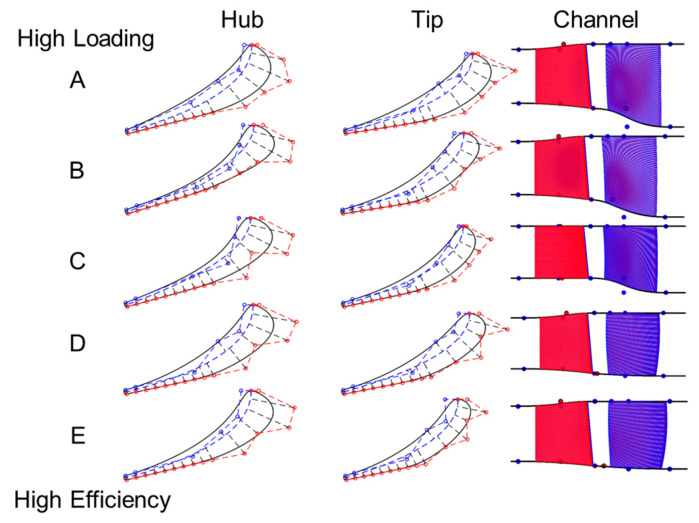
Stator optimal profiles and channel geometry.

Profiles of the stators along the pareto front are compared in Figure 13 Profile A has the highest stage loading of 2.26 with an efficiency of 91.3%, the degree of reaction is 0.33. Profile E shown at the bottom of Figure 13 is the highest efficiency design and has a maximum efficiency of 93.3%, stage loading of 1.94, and a degree of reaction of 0.54. Highly loaded stators feature high turning from hub to tip. The more efficient have less turning at the tip and their profiles are smaller. Additionally, as designs go towards higher efficiency, the stagger angle decreases with decreases turning from 58 to 55 deg and the suction side wedge angle decreases from 5 to 2 degrees. The channel shown on the right of Figure 13 flow moves from left to right. The red represents the stator and blue, the rotor. The channel has a high expansion ratio for highly loaded designs, i.e., Design A has an initial expansion ratio of 1.2 from stator inlet to stator exit, and from stator exit to rotor exit the ratio is 1.28. The expansion ratio decreases as one moves towards designs of higher efficiency.

**Figure 14:**
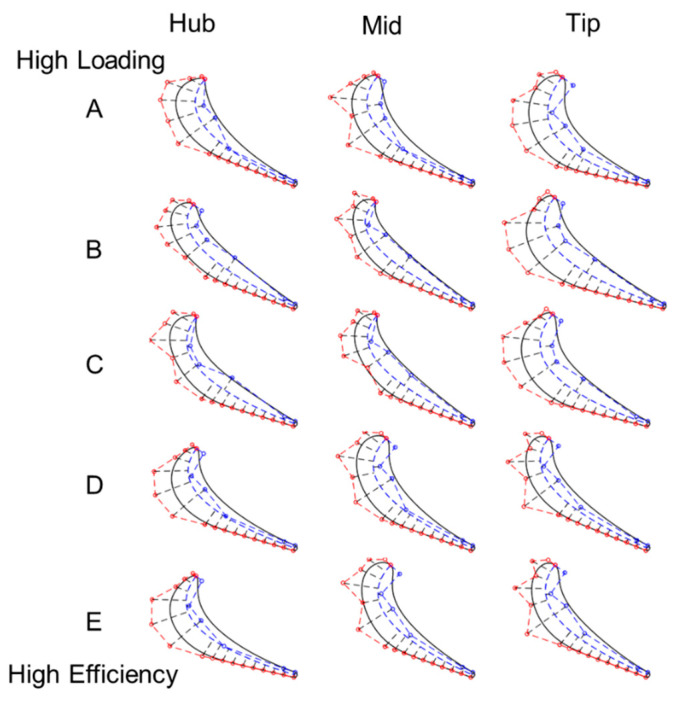
Rotor optimal profiles.

The highest loading design, profile A, is displayed in Figure 14-top and shows a larger change in thickness as opposed to profile E. The thickness of both the stator and rotor blades increases from hub to tip and so does the turning. 

Figure 14 compares rotor designs along the pareto front. Design A has the most loading, and it turns 19.5 kg/s of air 125 degrees through the turbine extracting 3.6MW. The maximum efficiency geometry, design E, turns 22.5 kg/s of flow 92 degrees and extracts 3.52MW. Design A’s profile is thicker near the tip, and β_3_ also increases from 66 to 74 deg at tip. However, β_2_ decreases from 40 deg at hub to 38 at the tip. Higher loading designs typically feature fatter profiles at the tip with more turning. More efficient designs on the other hand, have higher turning at the hub and less at the tip. In design E, the inlet metal angle β_2_ varies along the span from 38 to 43 to 38 degrees at the tip. β_3_ shows a different trend. It decreases from the hub to midspan, 73 to 65 degrees and slightly increases at the tip to 66 deg. In contrast, the suction side wedge angle fluctuates from 3 to 5 deg, then back to 3 deg. at the tip.

**Figure 15:**
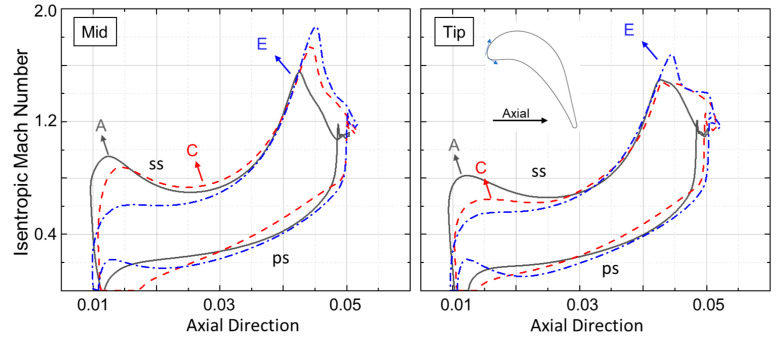
Rotor Isentropic Mach number. **Left**: Mid span. **Right**: Tip.

Isentropic Mach plots provides a precise non-dimensional estimation of the airfoil loading. Figure 15 compares the isentropic Mach number at the rotor midspan with the tip for 3 designs along the pareto front. Highly loaded designs have increased loading near the front of the blade followed by diffusion and re-acceleration of the flow until it leaves the trailing edge. This is true for both hub and tip. Designs with less stage loading are less front loaded and more aft-loaded, and have higher exit Mach numbers and large diffusion near the trailing edge.

## 4. Conclusions

Turbomachinery design and optimization are essential towards reducing specific fuel consumption in gas turbine engines. Modern design strategies combine 1D mean-line solvers, 2D through-flow analysis, followed by full 3D CFD to design the individual blades. The current paper describes a methodology that skips 2D through-flow analysis and dives directly into the design and optimization of a 3D turbine stage. The example presented was for the Purdue Experimental Turbine Aerothermal Lab. The methodology uses a fast 1D mean-line optimization to identify the optimal operating characteristics and provide the target stage flow angles. This procedure was followed by a full 3D parameterization and optimization of the stator, rotor, and channel geometry using a total of 75 design parameters. The aerodynamic efficiency of the turbine was simultaneously maximized with the stage loading, generating a Pareto front of prime turbine designs.

The optimization yielded a wide variety of possible designs, allowing for the investigation of loss-generation in both the stator and rotor. The stator losses reached values up to 12% for turning higher than 76 degrees, however, they can be kept as low as 6–7% if one limits the turning and maintains a degree of reaction above 0.4. Additionally, the lowest losses were observed for the geometries combining the minimum constrained stagger angle of 55 deg. with high pitch-to-chord ratios (up to 0.9) in a regime of low turning. 

A proper optimization of the rotor blade design can limit the loss generation to 10% for turning angles up to 110 degrees. From this point on, the losses increase significantly up to 15% for a turning angle of 125 deg. Furthermore, the majority of the rotor blade profiles along the Pareto front adopt a pitch-to-chord ratio of 0.7 to 0.75, stagger angles between 40 and 45 degrees, and feature a lean of the tip section towards the direction of rotation.

Using the database of possible designs, the mechanical efficiency was compared with the Horlock efficiency, computed using kinetic losses from stator and rotor. The comparison revealed a mismatch in the trend due to the rotor exit Mach numbers. A correction to the Horlock equation was proposed using the trends of stator and rotor exit Mach number which improved the accuracy for the turbine performance estimation from aerothermal measurement data to ±0.01%.

## Figures and Tables

**Table 1:** Stator and rotor grid sensitivity configuration. (Stator/Rotor).

Stator/Rotor	Mesh Size	Spanwise Divisions	Suction Side	Pressure Side
Coarser	1.5/1.5M	81/81	133/177	65/65
Coarse	2.4/2.2M	97/97	161/213	81/81
Fine	3.4/4.4M	117/141	193/257	97/97
Finer	5.6/7.0M	141/169	233/309	113/117

**Table 2:** Optimizer constants for both 1D and 3D optimizations.

	Symbol	Value
Mutation scale factor	F	0.6
Mutation rate	C	0.8

**Table 3:** Optimizer constants for both 1D and 3D optimizations.

	1D	3D
# Parameters	7	75
Design of Experiments	80	256
Population size	40	30
Populations Evaluated	35	15

**Table 4:** Characteristics of the optimal 1D Design.

Flow Angles		Performance			Mach Number	
α_2_	73	Power	MW	3.64	M_2_	0.89
α_3_	−33	Massflow	kg/s	22.8	M_2r_	0.34
β_2_	39.3	Degree of Reaction	-	0.39	M_3_	0.48
β_3_	−67.7	Stage Loading	-	1.74	M_3r_	1.04
-	-	T_01_	K	676	-	-

## Nomenclature

c	Chord [mm]
C_p_	Specific heat [J/(kgK)]
H	Blade height [mm]
h	Specific enthalpy [J/kg]
M	Mach number
P	ressure [bar]
r_p_	Degree of reaction
RPM	Rotational speed
RTZ	Radius, Tangential, Axial
s	Specific Entropy [J/(kgK)]
T	Temperature [K]
**Subscripts**	
0	Total flow quantity
1	Inlet
2	Rotor-stator interface
3	Outlet
ax	Axial component
θ	Tangential component
r	Radial component
s	Isentropic change across one row
ss	Isentropic change across two rows
**Greek symbols **	
α	Absolute flow angle
β	Relative flow angle
γ	Ratio of specific heats
η	Efficiency
ψ	Stage loading
ω	Angular velocity
